# Metagenome and Metabolic Pathways in Plaque Biofilms of Thai ELANE-Associated Neutropenic Patients: An Original Study and Scoping Review

**DOI:** 10.1055/s-0046-1818559

**Published:** 2026-03-25

**Authors:** Wanna Chetruengchai, Kanokwan Sriwattanapong, Chawan Manaspon, Kausar S. Fakhruddin, Lakshman Samaranayake, Vorasuk Shotelersuk, Thantrira Porntaveetus

**Affiliations:** 1 Center of Excellence for Medical Genomics, Department of Pediatrics, Faculty of Medicine, Chulalongkorn University, Bangkok, Thailand; 2 Excellence Center for Genomics and Precision Medicine, King Chulalongkorn Memorial Hospital, the Thai Red Cross Society, Bangkok, Thailand; 3 Center of Excellence in Precision Medicine and Digital Health, Department of Physiology, Faculty of Dentistry, Chulalongkorn University, Bangkok, Thailand; 4 Biomedical Engineering Institute, Chiang Mai University, Chiang Mai, Thailand; 5 Faculty of Dentistry, The University of Western Australia, Nedlands, Western Australia, Australia; 6 Faculty of Dentistry, University of Saskatchewan, Saskatchewan, Canada; 7 Faculty of Dentistry, University of Hong Kong, Hong Kong; 8 FutureDent Digital Center, Chulalongkorn University Implant and Esthetic Center, Faculty of Dentistry, Chulalongkorn University, Bangkok, Thailand

**Keywords:** periodontal diseases, oral microbiome, neutropenia, metagenomics, healthcare access, precision medicine

## Abstract

**Objective:**

Congenital neutropenia, particularly ELANE-associated forms, is associated with recurrent oral infections and aggressive periodontitis. While ELANE deficiency compromises oral health, its relationship to plaque biofilm ecology and metabolic function remains unclear. The oral microbiome-metabolome interplay in this condition remains largely uncharacterized globally. Here, we address this gap by characterizing the dental plaque metagenome and inferred metabolic pathways in a defined cohort of Thai neutropenia patients.

**Materials and Methods:**

In this exploratory study, we sequenced dental plaque samples from a defined cohort of nine individuals: three patients with severe congenital neutropenia or cyclic neutropenia (CyN) with confirmed ELANE variants, and six from age- and gender-matched healthy controls. Shotgun metagenomics was used for genomic analysis, followed by comprehensive microbiota examination. Subsequently, MetaCyc, a curated database, was used for in silico analysis and comparisons of the predicted functional pathways between the test and control plaque biofilms.

**Results:**

The principal coordinate analysis plot and heat map revealed distinct segregation of microbial profiles between the patients and control groups. A significant variation in the proportions of the five core phyla was noted in patients and controls. Two commensal species, Aggregatibacter sp oral taxon 458 and Leptotrichia sp oral taxon 212, were enriched in the controls. Conversely, four species were significantly enriched in the patients, Selenomonas flueggei, Streptococcus milleri, Kingella oralis, and Actinobaculum sp oral taxon 183; the latter being notably elevated across all patients. The MetaCyc in silico analyses suggested predicted enrichment of functional pathways associated with inflammation and oxidative stress in patients, including L-methionine biosynthesis IV, formaldehyde assimilation III, L-rhamnose degradation, and the superpathway of (R,R)-butanediol biosynthesis pathways.

**Conclusion:**

Our study advances the understanding of ELANE-associated periodontitis by moving beyond descriptive microbiota analysis to suggest potential associations between host immune deficiency, microbial dysbiosis, and the microbiota-associated metabolic pathway alterations. These findings provide preliminary insights into targeted periodontal care in neutropenic patients, though further validation in larger cohorts is required.

## Introduction

Neutrophils are the primary defense against microbial infections in humans and key leukocytes in the innate immune system. In the oral cavity, they are essential for maintaining an eubiotic ecosystem and preventing diseases such as periodontal infections. 1 2 Polymorphonuclear neutrophils (PMNs) are the innate immune system's first line of defense, maturing from granulocyte-monocyte progenitors in the bone marrow under the regulation of granulocyte-colony stimulating factor (G-CSF). 3 The ELANE gene (OMIM ^*^ 13130), which encodes the serine protease neutrophil elastase, is essential for this developmental process. Variants in ELANE can induce the unfolded protein response in myeloid precursors, causing a failure of PMN maturation and premature apoptosis. This maturation arrest leads to severe congenital neutropenia (SCN, MIM #202700) or cyclic neutropenia (CyN, MIM #162800), rare disorders with a combined prevalence of three to eight cases per million births. 4

While SCN and CyN differ in their clinical presentation and serial absolute neutrophil count (ANC) patterns, both phenotypes are characterized by profound neutropenia (<0.5 × 10 ^9^ /L), recurrent infections, organ dysfunction, and a heightened risk of leukemic transformation. 5 Currently, most patients are managed with G-CSF therapy, which helps elevate neutrophil counts and reduce the frequency of severe infections. 5 6

The interaction between the host immune system and the oral microbiota is critical for maintaining periodontal health. Periodontitis is a chronic, multifactorial inflammatory disease initiated by the accumulation of a dysbiotic plaque biofilm. 7 However, the ultimate destruction of gingiva, periodontal ligaments, and alveolar bone is largely mediated by the host's dysregulated immune-inflammatory response to these microbial challenges. 8 9 Under homeostatic conditions, neutrophils maintain the ecological balance of the gingival crevice. In patients with ELANE pathogenic variants, the quantitative and qualitative defects in neutrophils compromise this surveillance, rendering them highly susceptible to severe gingivitis and aggressive, early-onset periodontitis despite standard hygiene measures. 10 11

Microbiome findings in ELANE neutropenia are inconsistent. A recent 16S sequencing study found no difference in subgingival plaque microbiota between SCN patients and sex-matched controls, 12 whereas an earlier study reported skewed pathogenic proportions. 10 The dental plaque microbiota in ELANE variants remains poorly characterized, particularly at the metagenomic and metabolomic levels.

Moreover, many oral microbiome studies rely on saliva or subgingival samples, which reflect either a composite oral signal or a late-stage periodontal disease niche. 13 14 15 In contrast, supragingival plaque constitutes a site-specific, structured biofilm that is associated with neutrophil influx and the initiation and progression of periodontal inflammation. 16 Shotgun metagenomic analysis of dental plaque, therefore, enables high-resolution profiling of both taxonomic composition and functional metabolic potential within this critical ecological niche. 17 18

Accordingly, the central hypothesis of this study is that ELANE-associated neutropenia is linked to alterations in dental plaque microbial composition and predicted metabolic pathways, reflecting dysbiosis and functional shifts rather than direct mechanistic host–microbe causality. To test this hypothesis, this study characterized the plaque microbiome and metabolome in patients with ELANE variants and age- and sex-matched healthy controls using shotgun metagenomics and compared predicted functional pathways in silico via MetaCyc pathways analysis.

To contextualize our findings, we conducted a scoping review of published literature on oral or periodontal phenotypes in ELANE-related neutropenia, incorporating microbial and functional data where available. This synthesis allowed alignment of our results with existing evidence and identification of both consistent and novel patterns unique to our cohort.

## Materials and Methods

### Subjects and Sample Collection

Three patients with SCN or CyN and confirmed ELANE variants 6 were recruited from the Faculties of Medicine and Dentistry, Chulalongkorn University, Bangkok, Thailand. All procedures performed in studies involving human participants were in accordance with the ethical standards of the institutional and/or national research committee and with the 1964 Helsinki Declaration and its later amendments or comparable ethical standards. The study was approved by the Institutional Review Board, Faculty of Medicine, Chulalongkorn University (IRB 316/63) and was conducted in accordance with the 1964 Helsinki Declaration and its subsequent amendments. Written informed consent was obtained from all participants or their legal guardians.

Six age- and gender-matched healthy controls (two per patient) with no history of oral, periodontal, or systemic disease and no medication served as comparators. All participants were caries-free or had completed treatment ≥ 6 months before sampling. The patient group comprised a 6-year-old boy (SCN), a 7-year-old girl (CyN), and a 26-year-old woman (SCN), all receiving granulocyte colony-stimulating factor (G-CSF) therapy. Healthy controls included two boys and two girls aged 6 to 7 years, and two women aged 26 years. Due to the rarity of ELANE-associated neutropenia, recruitment was limited to three cases.

Supragingival and subgingival plaque from buccal and lingual surfaces of posterior teeth was collected using a sterile curette by a single examiner (TP), pooled in 1.5 mL tubes, and stored at −80°C.

### DNA Extraction and Metagenomics Sequencing

DNA was extracted from dental plaque biofilm using the QIAamp DNA Microbiome Kit (Qiagen, Hilden, Germany) according to the manufacturer's protocols and subjected to shotgun metagenomics sequencing using the TruSeq Nano DNA library prep and Illumina MiSeq with 2 × 300 bp read lengths (Macrogen, Seoul, South Korea). The nine samples generated 18.3 GB of raw sequencing data, averaging 2.0 GB per sample; this output reflects comparable sequencing depth among the samples.

### Microbial Profiling and Statistical Analysis

Raw sequencing reads were subjected to quality control and host-read removal using KneadData (version 0.7.4), which integrates Trimmomatic (version 0.36) 19 and Bowtie2 (version 2.4.5) 20 with default parameters for alignment to the human genome (hg19). Taxonomic analyses focused on microbial species with a relative abundance ≥ 0.1% in at least one sample to reduce potential artifacts while retaining sensitivity to low-abundance but biologically relevant taxa.

Taxonomic assignment and abundance estimation were performed using MetaPhlAn 3.0 with default parameters. 21 Taxonomic profiles were stratified into phylum, genus, and species levels using the hclust2.py script. Alpha diversity was assessed using Shannon diversity indices, and β diversity was evaluated by principal coordinates analysis (PCoA) and hierarchical clustering based on Bray–Curtis dissimilarity using STAMP and R. Group differences were assessed using permutational multivariate analysis of variance (PERMANOVA). Species-level relative abundances were visualized using heatmaps and descriptive abundance plots. Due to the limited sample size, effect sizes and confidence intervals were not calculated.

To identify differentially abundant biomarkers, Linear Discriminant Analysis Effect Size (LEfSe) was applied to both taxonomic and functional pathway data. Significance was defined as a *p* -value < 0.05 and a logarithmic LDA score > 2.0. LEfSe was used as a hypothesis-generating tool without formal multiple-testing correction. Significant results were visualized using bar plots and cladograms.

### Functional Analysis and Examination of Microbial Metabolome using MetaCyc Metabolic Pathway Database

The differential pathways between patient and control groups were analyzed using HUMAnN3, which aligns sequencing reads to the UniRef90 databases to quantify gene families and reconstruct MetaCyc metabolic pathways. MetaCyc is a comprehensively curated database of experimentally elucidated metabolic pathways ( https://metacyc.org/). ^22^ Unmapped and unintegrated pathways were excluded from downstream analyses. LEfSe was used to identify differentially abundant MetaCyc pathways ( *p* < 0.05; LDA score > 2) as a hypothesis-generating approach without formal multiple-testing correction. For normalization, MetaPhlAn taxonomic profiles were analyzed as relative abundances, while HUMAnN3 functional pathway abundances were normalized to copies per million to account for variations in sequencing depth

### Scoping Review of the Literature

To contextualize our metagenomic and metabolomic findings in ELANE-associated neutropenia, we conducted a scoping review aligned with the PRISMA extension for scoping reviews (PRISMA-ScR): checklist and explanation (2018) 23 and structured using the population-concept-context approach.

–Population: patients with genetically confirmed ELANE variants and SCN or CyN.
–Concept: oral/periodontal manifestations and microbiota composition (culture-based, 16S rRNA, or shotgun metagenomics) with functional/metabolomic analyses.
–Context: human studies (case reports, series, cohorts) published January 2000 to May 2024 reporting oral phenotypes with ELANE variants.

Search strategies, databases, and eligibility criteria are provided in Supplementary Table S1 (available in the online version only).

## Results

### Clinical Characteristics and Genetic Variants

Patient 1, a 7-year-old girl, developed multiple infections (otitis media, mastoiditis, sinusitis, scalp abscess, gastroenteritis) from 1 month of age. Diagnosed with SCN at 10 months, she received G-CSF (5 µg/kg, 4 ×/week), improving ANC from 345 ± 14 to 1,786 ± 59 cells/µL, though infections persisted. Oral exam at 8 years revealed early-onset periodontitis, oral ulcers, tooth mobility, and clinical signs of mucosal infection ( Fig. 1A ). At age of 8 years, all deciduous teeth were lost due to tooth mobility except one upper canine and one lower second molar, both with severe recession and bone loss; permanent first molars showed furcation involvement ( Fig. 1A, B ). Exome sequencing revealed a de novo heterozygous 12-bp in-frame insertion, c.289_300dup, p.(Gln97_Ala100dup) in the ELANE exon 3.

**Fig. 1:**
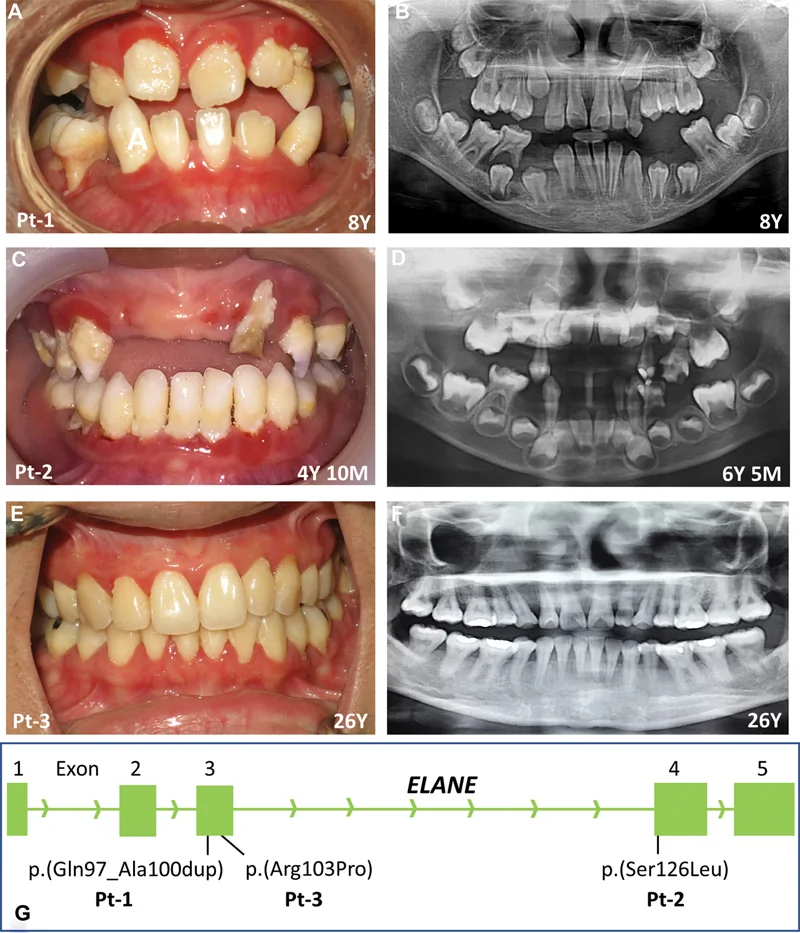
Orodental phenotype and genotypes of the patients. ( **A, B** ) Pt-1 (7-year-old girl) showed generalized periodontal inflammation, tooth mobility, malocclusion, alveolar bone loss, and furcation involvement. ( **C, D** ) Pt-2 (6-year-old boy) showed alveolar bone destruction and early loss of deciduous teeth. ( **E, F** ) Pt-3 (26-year-old woman) exhibited generalized horizontal bone loss. ( **G** ) Schematic diagram showing the mutation identified in the patients: p.(Gln97_Ala100dup) in Pt-1, p.(Ser126Leu in Pt-2, and p.(Arg103Pro) in Pt-3.

Patient-2, a 6-year-old boy, was diagnosed with CyN at age 6 (onset at 8 months). He experienced monthly colds, was treated with cefixime every 3 weeks, did not receive G-CSF, and had ANC 1 to 8 × 10 ^3^ /µL depending on cycle. Oral exam revealed early-onset periodontitis, characterized by generalized gingival inflammation and early loss of deciduous teeth ( Fig. 1C ); by age 6, only canines and two second molars remained, both with severe bone loss ( Fig. 1D ). Sequencing identified a de novo heterozygous missense variant, c.377C > T, p.(Ser126Leu) in ELANE exon 4.

Patient-3, a 26-year-old woman, was diagnosed with SCN at 7 months (onset at 2 weeks). She received G-CSF (6.5 µg/kg twice weekly), increasing ANC from 175 ± 18 to 968 ± 125 cells/µL. Oral examination revealed generalized periodontitis with marked gingival inflammation ( Fig. 1E ), and panoramic radiographs showed horizontal bone loss ( Fig. 1F ). Sequencing revealed a de novo heterozygous variant, c.308G > C, p.(Arg103Pro) in ELANE exon 3.

Full medical and genetic findings of the foregoing three patients have been reported in a previous study of ours. 6

### Microbial Profiles of Dental Plaque Biofilm in ELANE Patients and Controls

From nine plaque samples (patients = 3, controls = 6), we identified eight bacterial phyla, 17 classes, 26 orders, 35 families, 54 genera, and 162 species. For visualization purposes, Fig. 2A–C depicts the average relative abundance of taxa exceeding a 1% threshold. Beta diversity analysis showed significant differences between patients and controls; 114 species were shared, while 28 and 20 were unique to patients and controls, respectively ( Supplementary Material 1 , available in the online version only).

**Fig. 2:**
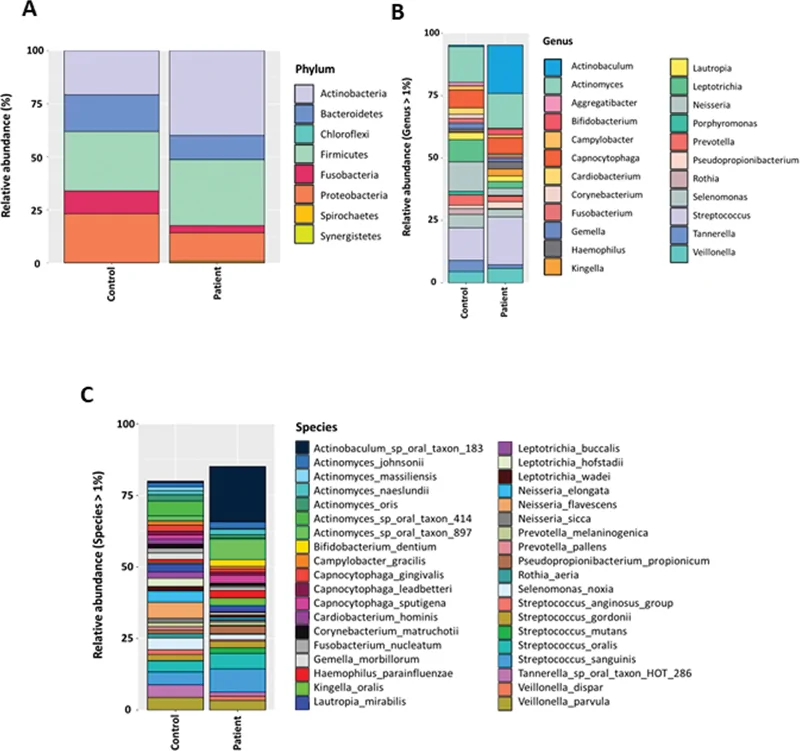
Microbial composition in patients and controls. ( **A** ) An average relative abundance of microbial phyla. ( **B** ) An average relative abundance of genera (>1%). ( **C** ) The average relative abundance of species (>1%).

In patients, Actinobacteria predominated, accounting for 35.60, 44.80, and 39.40% of the microbial community in the three individual samples (patients 1, 2, and 3, respectively), followed by Firmicutes (32.10, 33.10, and 28.00%) and Proteobacteria (13.10, 20.70, and 5.90%; Fig. 2A ). At the genus level, Actinobaculum (17.20, 22.70, and 18.20%), Streptococcus (30.40, 7.00, and 19.30%), and Actinomyces (10.80, 17.30, and 13.80%) were dominant across patient samples. At the species level, Actinobaculum sp. oral taxon 183 was consistently abundant in all patients (17.20, 22.70, and 18.20%), followed by Streptococcus sanguinis (2.60, 2.70, and 19.30%), Actinomyces sp. oral taxon 897 (13.20, 3.60, and 4.60%), Streptococcus oralis (8.90, 2.20, and 5.10%), and Veillonella parvula (4.60, 5.30, and 0.00%). In controls, Firmicutes dominated (28.04 ± 12.05%), followed by Proteobacteria (23.07 ± 16.36%) and Actinobacteria (20.78 ± 6.67%). The top genera were Actinomyces (14.29 ± 8.14%), Streptococcus (13.07 ± 8.63%), and Neisseria (11.86 ± 12.36%). Dominant species included Neisseria flavescens (5.65%), Actinomyces sp. oral taxon 414 (5.25%), Streptococcus sanguinis (4.52%), Tannerella sp. HOT 286 (4.41%) and Veillonella parvula (4.38%; Fig. 2A–C ).

In quantitative terms, a total of 114 out of 162 species were identified in both patients and controls. Twenty species were exclusively found in controls, and 28 species in patients ( Fig. 3A , Supplementary Material 1 , available in the online version only). Alpha diversity (Shannon index) did not differ significantly ( *p* = 0.26; Fig. 3B ), indicating similar richness and evenness in both groups, but PCoA revealed distinct community structures (β diversity; Fig. 3C ). The total diversity captured by the top two principal coordinates was 66.5%.

**Fig. 3:**
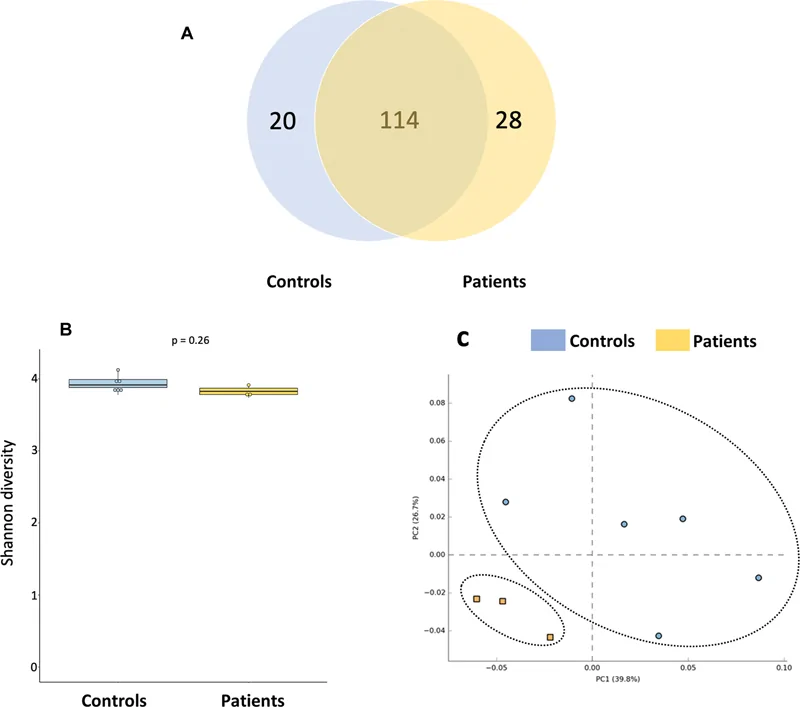
Diagrams showing the number of species and α- and β-diversity. ( **A** ) A Venn diagram representing the number of unique and shared species in patients and control groups. ( **B** ) Alpha diversity analysis, assessed by Shannon diversity, did not show a significant difference between patient and control groups. ( **C** ) Principal coordinate analysis (PCoA) of β-diversity based on Bray-Curtis dissimilarity demonstrated segregation between patient and control samples. ( **D** ) Hierarchical clustering of the top 20 differentially abundant bacterial species based on Bray–Curtis distance. Patient-1, -2, and controls-1, -2, -3, -4, who were children with mixed dentitions, were clustered and separated from patient-3, control-5, -6, who were adults with permanent dentition.

**Figure:**
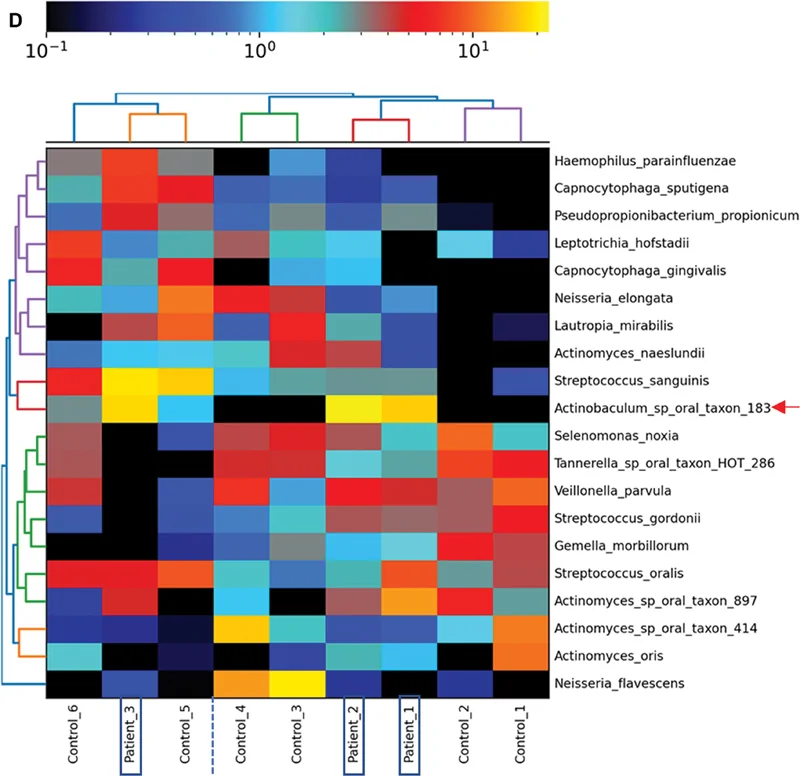


Heat map clustering grouped samples by age and dentition. Children (patients 1 and 2, control 1–4) with mixed dentition, and adults (patient 3, control 5–6) with permanent dentition ( Fig. 3D ). Tannerella sp. HOT 286 was present in all mixed-dentition controls, while permanent-dentition controls showed Capnocytophaga gingivalis, Streptococcus oralis, and S. sanguinis. Actinobaculum sp. oral taxon 183 dominated all patient samples and showed a markedly higher relative abundance compared with controls (0–2.76%; Fig. 3D ).

LEfSe analysis identified the microbial taxonomic differences between controls and patients ( Fig. 4A ). At the species level, four species were significantly enriched in patients ( *p* < 0.05; LDA score (log 10) > 3.6), and these comprised Actinobaculum sp oral taxon 183, Selemonas flueggei, Streptococcus milleri, and Kingella oralis. Two species comprising Aggregatibacter sp oral taxon 458 and Leptotrichia sp oral taxon 212 were significantly enriched in controls (LDA > 3.6). At the genus level, the genera Actinobaculum (LDA score > 4.8) and Kingella (LDA > 3.6) were enriched in patients, while the genus Aggregatibacter was enriched in controls. At family, order, class, and phylum levels, Actinomycetaceae, Actinomycetales, Actinobacteria, and Actinobacteria, respectively, were significantly abundant in patients (LDA > 4.8; Fig. 4A ). Cladogram analysis confirmed phyla Actinobacteria abundance in patients compared with controls ( Fig. 4B ).

**Fig. 4:**
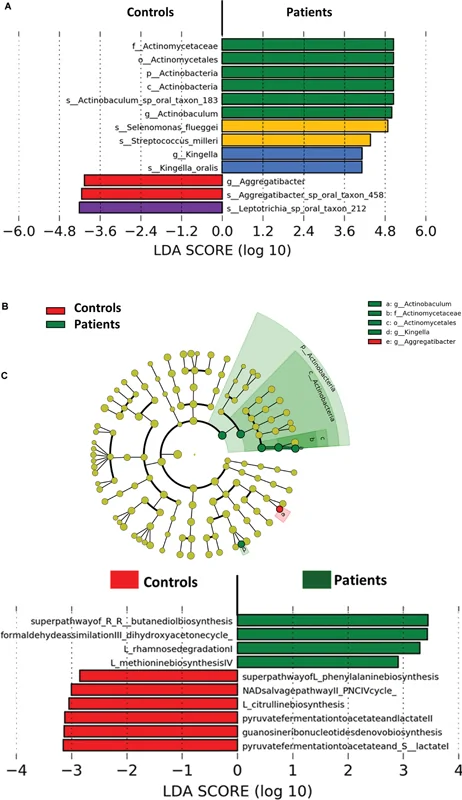
Linear discriminant analysis Effective Size (LEfSe) plot. The LEfSE showed differential abundance of microbial taxa between patient and control groups, visualized by a bar plot of the LDA score (log 10) ( **A** ) and cladogram ( **B** ). The left panel of the bar plot indicated significantly more abundant microbes in controls, and the right panel indicated significantly higher abundance of microbes in patients. Cladogram exhibited that the phylum Actinobacteria was enriched in patients ( **C** ), the LEfSe plot showing the differential MetaCyc pathways between patient and control groups.

### Microbial Functional Genes and Pathways in Patients and Controls

To assess the potential functionality of dental plaque biofilm microbiota, microbial genes were annotated and analyzed using HUMANN3 based on the MetaCyc database. Three hundred and nineteen pathways were detected in patients versus 301 in controls. Most were biosynthetic pathways, followed by degradation/utilization/assimilation pathways, and generation of precursor metabolites and energy pathways ( Supplementary Material 2 , available in the online version only).

For the biosynthesis pathway, the cofactor, carrier, and vitamin biosynthesis sub-pathway classes were the most abundant, followed by the amino acid biosynthesis and the nucleoside and nucleotide biosynthesis classes, respectively. As for the degradation/utilization/assimilation pathway, patients showed more carbohydrate degradation and carboxylate degradation; controls had more inorganic nutrient metabolism. For the generation of precursor metabolites and energy pathways, the fermentation was the most abundant class, followed by the tricarboxylic acid (TCA) cycle and the glycolysis classes, respectively ( Supplementary Material 3 , available in the online version only).

Ten pathways differed significantly (LDA > 2): patients were enriched in (1; R,R)-butanediol biosynthesis, (2) formaldehyde assimilation III, (3) L-rhamnose degradation, and (4) L-methionine biosynthesis IV. Controls were enriched in (1) pyruvate fermentation to acetate and (S)-lactate I, (2) guanosine ribonucleotide biosynthesis, (3) pyruvate fermentation to acetate and lactate II, (4) L-citrulline biosynthesis, (5) NAD salvage pathway II, and (6) L-phenylalanine biosynthesis ( Fig. 4C ; Supplementary Materials 4 and 5 , available in the online version only).

### Clinical, Genetic, Microbial, and Functional Insights into ELANE-Associated Periodontal Disease: A Scoping Review and Thai Cohort Comparison

To contextualize our findings, we reviewed seven studies 10 24 25 26 27 28 29 published between 2011 and 2024, comprising 26 patients with confirmed ELANE variants and oral manifestations. We compared these with our Thai cohort of three patients and six age-matched healthy controls ( Table 1 ).

**Table 1:** Summary of clinical, genetic, microbial, and functional findings in reported cases with ELANE variants

Study (y), country	Population ( *n* ), age, gender	Clinical periodontal status	ELANE mutation status	Methodology (sequencing/analysis)	Key microbial findings	Metabolomic/functional pathways
Ye et al (2011) Sweden	14 patients (13 with SCN, 1 with CyN) Age range: 6–50 y Gender: NR	ELANE mutation group ( *n* = 6): ⅚ had periodontitis or were edentulous due to periodontitis. CyN patient ( *n* = 1): healthy periodontium. Common findings: chronic gingivitis, alveolar bone loss, and early tooth loss in severe cases.	ELANE mutations ( *n* = 6): PN1: D89H PN2: C122S PN3: C26S PN4: C26S PN5: C26S PN9: L92H	Microbiota analysis: 16S rDNA pyrosequencing of subgingival samples. Cytokine/peptide analysis: Luminex immunoassay for IL-1β, IL-6, IL-17, and TNF-α in gingival crevicular fluid and plasma. Western blotting for antimicrobial peptides (HNP1–3, pro-LL-37/LL-37).	Dominant genera: Fusobacterium, Streptococcus, Prevotella, Treponema. ELANE mutation group: Skewed microbiota similar to periodontitis references (enriched Fusobacterium, Prevotella, Treponema)	Neutrophil dysfunction: ELANE mutations impair neutrophil elastase, leading to defective antimicrobial peptide (e.g., LL-37, HNP1–3) production and apoptosis of precursors. G-CSF pathway: patients with ELANE mutations required higher G-CSF doses but still had persistent periodontal disease
Shu et al (2015) China	7 Pediatric patients Median age at diagnosis: 1.5 y (range: 0.056–8.58 y). Median age at disease onset: 30 d (range: 20–120 d). 4 Males, 3 Females from 6 families	All patients exhibited stomatitis, chronic gingivitis, and osteopenia. Teeth were abnormally shaped and colored, with early loss of teeth reported. Infections (e.g., abscesses) were frequently pus-free	6 novel heterozygous mutations identified in ELANE: P1: Exon 5 deletion (g.8670delG; p.D201 fs X267). P2: Exon 5 substitution (g.8711C > A; p.G214E). P3: Exon 2 substitution (g.5717T > C; p.S67P). P4: Exon 3 deletion (g.6035–6049del; p.Q97–Y101del). P5: Exon 4 insertion (g.8312–8313ins15; p.I35–I36ins5). P6/P7: Exon 4 substitution (g.8372T > C; p.W156R). Mutations were sporadic; no carriers found in parents/siblings (except P6/P7, siblings with the same mutation but unaffected parents)	PCR and direct sequencing of ELANE genomic DNA and cDNA. TA cloning of cDNA to confirm heterozygous mutations. Bone marrow aspirates, peripheral blood counts, and immunological tests (e.g., antineutrophil antibodies) were performed	Fungal co-infections noted in oral/respiratory infections	Neutrophil elastase dysfunction: Mutations affected the mature protein, disrupting myeloid differentiation. G-CSF pathway: Treatment with granulocyte-colony stimulating factor (G-CSF) improved neutrophil counts but did not restore normal function
Aota et al (2019) Japan	1 patient (17-y-old) Female	Diagnosed with CyN shortly after birth. Severe periodontitis, tooth mobility, gingival inflammation (redness/swelling), alveolar bone resorption, apical lesions in mandibular lateral incisors	Mutation: novel heterozygous frameshift mutation in ELANE exon 4 (c.538delC, p.Leu180Ser fsX11). Inheritance: Shared with the patient's mother (who had milder CyN symptoms)	Genetic testing: PCR and direct sequencing Diagnostic criteria: Persistent neutropenia (ANC: 0–1400/μL, cyclical	Elevated levels of Prevotella intermedia, Porphyromonas gingivalis, Campylobacter rectus, Treponema denticola, and Tannerella forsythia compared with healthy controls	Proposed mechanism: ELANE mutation → misfolded neutrophil elastase → unfolded protein response → apoptosis of neutrophil precursors. Neutrophil deficiency exacerbates dysbiosis and periodontal destruction
Yang et al (2020) China	1 patient (4-y-old) Female	Symptoms began at age 2. Initial presentation: generalized tooth mobility, gingival bleeding, severe alveolar bone loss (posterior > anterior regions), recurrent oral ulcers, and poor oral hygiene (DI-S = 1). Follow-up (3 y): Persistent gingival inflammation, probing bleeding (+), progressive alveolar bone loss, and tooth mobility (classes I–II)	Mutation: heterozygous ELANE mutation (c.661G > T, exon 4). Pathogenic Mechanism: mutant neutrophil elastase likely triggers unfolded protein response and apoptosis of neutrophil precursors	Genetic testing: Sanger sequencing identified the ELANE mutation. Diagnostic Criteria: Based on Severe Chronic Neutropenia International Registry (SCNIR) guidelines: Persistent neutropenia (<0.5 × 10 ^9^ /L). Recurrent infections (e.g., pneumonia, gingivitis)	Not explicitly discussed (focus on genetic and clinical management)	Blood tests: persistent neutropenia (neutrophils: 0.11–0.410 9 /L). Elevated blood lead levels (600 μg/L initially, reduced to 288 μg/L posttreatment). Reduced antimicrobial peptide LL-37 due to neutrophil deficiency exacerbates periodontal infections
Lao et al (2022) China	1 patient 12-y-old) Female	Symptoms began at age 4. Initial Presentation: severe chronic periodontitis, generalized deep periodontal pockets (6–9 mm), Class II/III tooth mobility, Gingival inflammation, Recurrent aphthous ulcers (RAU), and pathological tooth migration	Novel mutations: c.180T > G (p.I60M) c.182C > G (p.A61G) Location: Exon 2 of ELANE	Bioinformatics analysis: mutations reduced protein stability (weakened hydrogen bonds) but did not alter catalytic affinity for substrates	Not explicitly studied (focus on genetic/molecular mechanisms)	Not explicitly discussed (focus was on clinical management). Pathogenic mechanism: mutant neutrophil elastase had impaired stability/integrity but normal catalytic function, suggesting misfolded protein-induced apoptosis of neutrophil precursors
Wang et al (2022) China	1 patient (30-mo-old) Male	Gingivitis Oral thrush (white curds in the mouth) Recurrent oral ulcers	Mutation: heterozygous de novo missense variant in the ELANE gene (c.125C > T, exon 2, chr19p13.3). Pathogenicity: Classified as pathogenic (ACMG criteria: PS1 + PS2 + PM2 + PP3). Parental Status: parents had normal wild-type ELANE (no mutation detected)	Whole-exome sequencing (mean 10 Gb data, >99% target coverage). Variant annotation: Chigene online system (MAFs from 1,000 Genomes, dbSNP, ESP, ExAC). Pathogenicity prediction: Provean, Sift, Polyphen2, MutationTaster, M-Cap, and Revel. Validation: Sanger sequencing confirmed a de novo mutation in the proband	Fungal thrush (Candida spp.) Positive Mp-DNA, MP-Ab-IgM, RSV-Ab-IgM, influenza B virus Ab-IgM	Proposed mechanism: mutant neutrophil elastase (NE) triggers apoptosis via unfolded protein response, impairing neutrophil maturation. Bone Marrow Findings: Myelodysplasia with maturation arrest (promyelocytes 8%, lobulated nuclei 2.5%)
Shu et al (2024) China	1 patient (2.5 y-old) Female	Recurrent gingivitis (since the patient was 7 mo old) Persistent mouth ulcers (since the patient was 6 mo old) Disordered and pigmented teeth	Mutation: deep intronic variant (c.598 + 79G > T) in ELANE Effect: aberrant splicing → insertion of 83 bp intron → premature termination codon (p.Gly200ValfsTer40) Inheritance: De novo (absent in parents and elder sister)	Sequencing: whole-exome sequencing (WES) initially negative Full-length ELANE gene sequencing (exons + introns) via Sanger cDNA sequencing + T-A cloning Functional assays: mini-gene splicing assay (confirmed aberrant splicing) Confocal microscopy (myeloperoxidase/neutrophil elastase expression)	Not explicitly studied (focus on genetic/molecular mechanisms).	Unfolded protein response (UPR): mutant ELANE → accumulation of misfolded elastase → ER stress → UPR activation → neutrophil apoptosis. Protein expression: elevated myeloperoxidase and neutrophil elastase in patient cells (confocal microscopy)
Thai study (2026)	9 participants (3 SCN/CyN patients with ELANE variants, 6 healthy controls) 3 patients: 6, 7, and 26 y Controls: 6–7 y (4 children), 26 y (2 adults)	Patients: severe gingivitis, early-onset periodontitis, tooth mobility, and alveolar bone loss. Controls: Healthy, no periodontal disease	Pt-1: c.289_300dup (p.Gln97_Ala100dup Pt-2: c.377C > T (p.Ser126Leu) Pt-3: c.308G > C (p.Arg103Pro)	Sequencing: shotgun metagenomics (Illumina MiSeq, 2 × 300 bp) Analysis: MetaPhlAn 3.0 (taxonomy), HUMANN3 (MetaCyc pathways), LEfSe (differential abundance)	Patients: enriched in Actinobaculum sp. oral taxon 183, Selenomonas flueggei, Streptococcus milleri, Kingella oralis Actinobacteria are dominant in patients. Controls: enriched in Aggregatibacter sp. oral taxon 458, Leptotrichia sp. oral taxon 212 Firmicutes are dominant in controls	Patients: upregulated L-methionine biosynthesis IV Formaldehyde assimilation III, L-rhamnose degradation (R,R)-butanediol biosynthesis Controls: upregulated pyruvate fermentation, NAD salvage, L-citrulline biosynthesis

Note: This table is descriptive in nature, and no statistical analyses were performed.

### Clinical Periodontal Presentations

Across studies, patients, mostly children, presented with early-onset gingivitis, severe periodontitis, tooth mobility, recurrent oral ulcers, and alveolar bone loss. 24 25 28 29 Additional features included abnormally shaped/pigmented teeth, osteopenia, 24 28 and oral thrush. 28 Notably, all had systemic neutropenia, confirmed by recurrent infections and persistently low ANC. 10 26 27 Our Thai cohort mirrored these findings, with all three patients exhibiting severe periodontal destruction, reinforcing the ELANE-Periodontitis link.

### Genetic Findings

The reviewed studies revealed a heterogeneous spectrum of ELANE variants, including missense variants (e.g., c.125C > T, c.377C > T), frameshift variants, exon deletions, and a rare deep intronic variant causing aberrant splicing. 24 25 26 27 29 Most variants were de novo, though one maternally inherited case was reported. 25 In our cohort, we identified three previously unreported ELANE variants: a 12-bp in-frame duplication (c.289_300dup; p.Gln97_Ala100dup) and two missense variants (c.377C > T; p.Ser126Leu and c.308G > C; p.Arg103Pro), expanding the genetic landscape of ELANE-associated dysbiosis.

### Microbial Findings

Four studies profiled microbiota using culture-based or 16S rDNA sequencing, 10 24 25 28 shown in Table 1 . These studies implicated classical periodontopathogens, such as Porphyromonas gingivalis, Treponema denticola, and Prevotella intermedia. 10 25 Ye et al further linked dysbiosis to reduced antimicrobial peptides, for instance, LL-37, HNP1–3. 10 Our shotgun metagenomics analysis extended these observations, revealing patient-specific enrichments of Actinobaculum sp. oral taxon 183, Selenomonas flueggei, Streptococcus milleri, and Kingella oralis, while controls harbored higher abundances of Aggregatibacter and Leptotrichia species. Strikingly, Actinobacteria dominated patient microbiomes, whereas Firmicutes prevailed in controls.

### Functional and Predicted Metabolic Pathways Analysis

Using HUMANN3 and MetaCyc, we identified enriched pathways in patients that are associated with inflammation and oxidative stress, including L-methionine biosynthesis IV, formaldehyde assimilation III, and (R,R)-butanediol biosynthesis. Conversely, control samples showed a higher abundance of genes related to pyruvate fermentation, NAD salvage, and L-citrulline biosynthesis pathways. These findings suggest that the microbial shifts observed in ELANE-associated patients are accompanied by differences in inferred metabolic potential, which may contribute to the periodontal environment.

## Discussion

This study builds on our earlier Thai ELANE cohort report, 6 by moving from genetic characterization to integrated oral microbiome and metabolic pathway analysis in the same rare patient group. Shotgun metagenomics and in silico functional profiling revealed species-level dysbiosis and distinct pro-inflammatory metabolic signatures, advancing from descriptive genotyping to mechanistic insights into host–microbe interactions. All three patients, spanning childhood to adulthood, showed severe periodontal destruction consistent with chronic gingival infection, which may be related to defective neutrophil function. 30 31 32

Shotgun metagenomic sequencing revealed distinct microbial profiles and altered proportions of five core phyla between patients and controls. Actinobacteria predominated in patients, followed by Firmicutes and Proteobacteria, while controls showed Firmicutes, Proteobacteria, and Actinobacteria, in descending order of dominance, consistent with healthy microbiomes. 33 34

Correspondingly, the top three genera in controls were Actinomyces, Streptococcus, and Neisseria, while Actinobaculum was the most predominant genus in patients, followed by Streptococcus and Actinomyces. At the species level, Neisseria flavescens was the most prominent in controls, while Actinobaculum sp. oral taxon 183 was the most dominant in the patient. These indicate microbial dysbiosis in patients and a hierarchical disparity in the phyla and genera in patients and controls. Alpha diversity did not differ significantly, suggesting similar richness despite compositional shifts.

Streptococcus sanguinis and Veillonella parvula were abundant in both groups. The former is a healthy biofilm pioneer, 35 yet a binding target for periodontopathogens such as P. gingivalis and F. nucleatum, while the latter is a prevalent plaque species. 36 Both taxa are considered as a bridging species within developing biofilms due to their distinct coaggregation interactions and abilities to stimulate the growth of other biofilm colonizers. Though V. parvula is also viewed by some as a precursor pathogen signaling periodontal disease onset. 37

Our analysis suggests that host developmental stage and dentition status (mixed vs. permanent) are associated with variations in microbial community structure, largely independent of disease status. 38 39 The clustering pattern observed in Fig. 3D is consistent with previously described changes in the oral ecosystem during maturation. We observed dentition-associated microbial patterns, including the detection of Tannerella sp oral taxon HOT 286 exclusively in healthy children with mixed dentition (4.5–7.9%). This observation suggests that the presence of this taxon may be related to the mixed dentition phase. Tannerella sp. HOT 286 is reported to require close association with other bacteria for growth 40 and shares approximately 50% of its genes with its closest phylogenetic relative, Tannerella forsythia, a species commonly associated with periodontal disease. However, Tannerella sp. HOT 286 differs from T. forsythia in gene and GC content, metabolic pathways, and the absence of several virulence-associated genes, including karilysin, prtH, and bspA. These differences have been proposed to underlie its comparatively lower pathogenic potential. 41

In contrast, control individuals with permanent dentition exhibited a microbiome profile characteristic of a more mature biofilm community, with Capnocytophaga gingivalis, Streptococcus oralis, and Streptococcus sanguinis among the predominant taxa. 42 These species are generally regarded as being associated with oral plaque biofilm stability. 43 Consistent with this, Belstrøm et al reported higher transcriptional activity of S. oralis and S. sanguinis in dental plaque from healthy individuals compared with periodontitis patients. 44

Crucially, however, distinct disease-associated signals emerged that appear to transcend these developmental strata. While health-associated taxa varied by age, the overabundance of Actinobaculum sp. oral taxon 183 was observed in both pediatric (mixed dentition) and adult (permanent dentition) patients relative to their respective controls. This suggests that while physiological maturation dictates the basal microbiome structure, the specific dominance of Actinobaculum sp. represents a dysbiotic signature likely driven by ELANE-associated neutropenia rather than developmental factors.

Taken together, these observations suggest that age and dentition status are associated with substantial microbiome heterogeneity, which should be considered when interpreting disease-related microbial patterns in cohorts spanning multiple developmental stages.

Two species, Aggregatibacter sp oral taxon 458 and Leptotrichia sp oral taxon 212, were exclusively found in the control sample and not in patient samples. It is notable that Aggregatibacter. sp oral taxon 458 is known to be predominant in healthy biofilm plaque. 45 On the other hand, Leptotrichia sp oral taxon 212 was found more in the oral microbiome of never-smokers compared with that of smokers, 46 suggesting that these two species may serve as surrogate indicators of a healthy eubiotic oral ecosystem.

In contrast, four species that were notably enriched in patient biofilm samples were Actinobaculum sp oral taxon 183, Selemonas flueggei, Streptococcus milleri, and Kingella oralis. Previous studies have noted that Kingella oralis, for example, is increased in the dental plaque of periodontitis patients 47 while Streptococcus milleri, a common oral commensal, acts as an opportunistic pathogen. 48 On the other hand, Actinobaculum sp oral taxon 183 was detected in the dental plaque of childhood caries. 49 All three of these organisms therefore have a pathogenic potential, and this, to some extent, is consistent with our findings.

Although α diversity (Shannon index) did not differ significantly between groups ( *p* = 0.26), compositional shift likely arises from taxon-specific abundance shifts rather than overall diversity loss. In contrast, β diversity differed significantly ( Fig. 3C ), indicating that neutrophil deficiency is associated with community structure and enriches pathobionts despite comparable richness. 32 Specifically, ELANE-associated neutropenia was characterized by distinct microbial assemblages enriched with pathobionts such as Actinobaculum sp. These findings suggest that functional shifts in the plaque biofilm may be attributed to altered community composition rather than reduced diversity.

MetaCyc analysis identified predicted enriched pathways in the patients, including L-methionine biosynthesis IV, formaldehyde assimilation III, and L-rhamnose degradation. The enrichment of the L-methionine biosynthesis IV pathway suggests a higher potential for methionine production, an amino acid often essential for the survival and virulence of various oral pathogens. 50 Similarly, the formaldehyde assimilation III (dihydroxyacetone cycle) was more abundant in patients. This pathway may reflect a microbial adaptation to oxidative stress. 51 Finally, the L-rhamnose degradation pathway that converts L-rhamnose to glycerin phosphate and (S)-lactaldehyde, and pyruvate, 52 is important for the metabolic activities of most bacteria. 53 Collectively, the differential abundance of these metabolic pathways suggests a functional potential that may be associated with microbial survival within the compromised immune environment of ELANE-associated patients. However, as these results are based on metagenomic inference, further metabolomic studies are necessary to validate the actual activity of these pathways.

### Clinical Relevance

Our findings provide preliminary evidence linking microbial shift with altered metabolic potential, offering a foundation for future precision-based care. The enrichment of Actinobaculum sp. and Kingella oralis identifies them as candidate biomarkers, which, upon further validation, could aid in the early detection of dysbiosis in this vulnerable population. Furthermore, the identification of predicted enriched pathways, such as those involved in biosynthesis and oxidative stress response, highlights potential avenues for therapeutic exploration, including the development of targeted pathway inhibitors or species-specific antimicrobial strategies. 54

### Review and Comparative Analysis of ELANE-Associated Periodontal Disease

Prior ELANE periodontitis studies largely reported genus-level shifts, for instance, Fusobacterium/Prevotella 10 or genetic mechanisms like UPR activation, 27 29 missing the species-level perturbations (e.g., Actinobaculum sp. oral taxon 183, 19.37%) and metabolic dysregulation we identify. Our integration of shotgun metagenomics and metabolomics reveals specific pathobiont enrichment and inflammatory pathway upregulation, offering actionable targets.

## Study Limitations

Several considerations should be taken into account when interpreting the findings of this study. The sample size was necessarily small because of the rarity of ELANE-associated neutropenia, which reduces statistical power. Although distinct microbial shifts were observed, the analysis was conducted in silico and may be insufficient to detect more subtle microbial or metabolic differences, and the generalizability of the findings remains limited. In addition, variability in age and dentition status among participants may act as a confounding influence. As indicated by the clustering analysis, developmental stage is associated with differences in the oral microbiome independent of disease status, complicating the attribution of microbiome features to disease alone. The cross-sectional study design further constrains interpretation, as temporal changes could not be evaluated, and the observed relationships are therefore associative rather than causal. Interpretation is also influenced by the absence of a nonneutropenic periodontitis control group, which limits differentiation between microbiome patterns related to neutrophil dysfunction and those associated with periodontal inflammation more broadly. For instance, the increased abundance of Actinobaculum sp. may reflect periodontal pocket formation common to multiple forms of periodontitis rather than a feature specific to ELANE variants. Moreover, functional pathway insights were derived from metagenomic inference rather than direct metabolomic measurements, and host immune parameters were not assessed to substantiate the proposed mechanistic interpretations. Finally, all participants received long-term G-CSF therapy as part of standard care. Given that G-CSF can influence immune responses and mucosal environments, the observed microbial patterns likely reflect an interplay between the underlying immunodeficiency and chronic pharmacological exposure rather than neutropenia alone.

## Conclusion

This study presents an integrated metagenomic and inferred metabolic pathway analysis of dental plaque in a defined cohort of Thai patients with ELANE-associated neutropenia. We identified a distinct microbial shift, characterized by an enrichment of pathobionts like Actinobaculum and predicted enrichment of metabolic pathways reflecting pro-inflammatory potential. These results suggest that impaired neutrophil surveillance is associated with a microbial environment consistent with the severe periodontitis observed in these patients. Although preliminary, these findings highlight putative microbial and predicted metabolic potential that warrant further investigation in larger, longitudinal studies incorporating host immune analyses to validate their utility as biomarkers or therapeutic targets.
