# Supplementary material for: Metagenome and Metabolic Pathways in Plaque Biofilms of Thai ELANE-Associated Neutropenic Patients: An Original Study and Scoping Review
Source: Eur J Dent. 2026 Mar 25;20(4):1281–96. doi: 10.1055/s-0046-1818559 (PMC13623484; doi:10.1055/s-0046-1818559)
Supplement: Supplementary file 1 [file 10-1055-s-0046-1818559-s2594489.pdf]

Metagenome and Metabolic Pathways in Plaque Biofilms of Thai ELANE-Associated Neutropenic Patients: An Original Study and Scoping Review

Supplementary Material 1: Species level shared between control and patient.

Group	Number	Species
Control and Patient	114	*Abiotrophia defectiva*
		Abiotrophia sp HMSC24B09
		Actinobaculum sp oral taxon 183
		Actinomyces georgiae
		Actinomyces hongkongensis
		Actinomyces johnsonii
		Actinomyces massiliensis
		Actinomyces naeslundii
		Actinomyces odontolyticus
		Actinomyces oris
		Actinomyces sp oral taxon 180
		Actinomyces sp oral taxon 414
		Actinomyces sp oral taxon 897
		Aggregatibacter aphrophilus
		Aggregatibacter segnis
		Aggregatibacter sp oral taxon 458
		Anaeroglobus geminatus
		Atopobium rimae
		Bacteroidetes oral taxon 274
		Bifidobacterium dentium
		Campylobacter concisus
		Campylobacter curvus
		Campylobacter gracilis
		Campylobacter showae
		Capnocytophaga gingivalis
		Capnocytophaga granulosa
		Capnocytophaga haemolytica
		Capnocytophaga leadbetteri
		Capnocytophaga ochracea
		Capnocytophaga sp oral taxon 332
		Capnocytophaga sp oral taxon 863
		Capnocytophaga sputigena
		Cardiobacterium hominis
		Cardiobacterium valvarum

		Catonella morbi
		Centipeda periodontii
		Corynebacterium durum
		Corynebacterium matruchotii
		Eikenella corrodens
		Eikenella sp NML130454
		Eubacterium brachy
		Eubacterium infirmum
		Eubacterium yurii
		Fusobacterium nucleatum
		Fusobacterium periodonticum
		Gemella haemolysans
		Gemella morbillorum
		Gemella sanguinis
		Granulicatella adiacens
		Haemophilus parainfluenzae
		Kingella denitrificans
		Kingella oralis
		Lachnoanaerobaculum saburreum
		Lautropia mirabilis
		Leptotrichia buccalis
		Leptotrichia hofstadii
		Leptotrichia sp oral taxon 212
		Leptotrichia sp oral taxon 215
		Leptotrichia sp oral taxon 225
		Leptotrichia sp oral taxon 498
		Leptotrichia sp oral taxon 847
		Leptotrichia sp oral taxon 879
		Leptotrichia wadei
		Megasphaera micronuciformis
		Neisseria bacilliformis
		Neisseria elongata
		Neisseria flavescens
		Neisseria macacae
		Neisseria mucosa
		Neisseria sicca
		Neisseria sp oral taxon 014
		Oribacterium sp oral taxon 078
		Ottowia sp oral taxon 894

		Parvimonas micra
		Porphyromonas somerae
		Porphyromonas sp oral taxon 278
		Prevotella denticola
		Prevotella intermedia
		Prevotella maculosa
		Prevotella melaninogenica
		Prevotella micans
		Prevotella nigrescens
		Prevotella oris
		Prevotella oulorum
		Prevotella pallens
		Prevotella saccharolytica
		Pseudopropionibacterium propionicum
		Rothia aeria
		Rothia dentocariosa
		Scardovia wiggsiae
		Selenomonas artemidis
		Selenomonas infelix
		Selenomonas noxia
		Selenomonas sp F0473
		Selenomonas sp FOBRC6
		Selenomonas sp oral taxon 138
		Selenomonas sp oral taxon 892
		Selenomonas sp oral taxon 920
		Selenomonas sputigena
		Slackia exigua
		Stomatobaculum longum
		Streptococcus anginosus group
		Streptococcus cristatus
		Streptococcus gordonii
		Streptococcus mutans
		Streptococcus oralis
		Streptococcus sanguinis
		Streptococcus sp oral taxon 056
		Tannerella sp oral taxon 808
		Tannerella sp oral taxon HOT 286
		Treponema maltophilum
		Treponema socranskii

		Veillonella atypica
		Veillonella parvula
Patient	28	*Actinomyces cardiffensis*
		Actinomyces sp oral taxon 448
		Anaerolineaceae bacterium oral taxon 439
		Bacteroidetes bacterium oral taxon 272
		Campylobacter rectus
		Capnocytophaga sp oral taxon 338
		Eubacterium minutum
		Eubacterium saphenum
		Fretibacterium fastidiosum
		Haemophilus sp HMSC71H05
		Lactobacillus fermentum
		Neisseria subflava
		Olsenella uli
		Porphyromonas endodontalis
		Prevotella loescheii
		Prevotella oralis
		Prevotella salivae
		Rothia mucilaginosa
		Selenomonas flueggei
		Selenomonas sp oral taxon 126
		Streptococcus massiliensis
		Streptococcus milleri
		Streptococcus sinensis
		Treponema denticola
		Treponema vincentii
		Veillonella dispar
		Veillonella infantium
		Veillonella sp T11011 6
Control	20	*Actinomyces sp oral taxon 170*
		Actinomyces viscosus
		Alloprevotella tannerae
		Atopobium parvulum
		Dialister invisus
		Dialister pneumosintes
		Eubacterium sulci
		Johnsonella ignava
		Lachnospiraceae bacterium oral taxon 096

		Mitsuokella sp oral taxon 131
		Mogibacterium diversum
		Mogibacterium timidum
		Olsenella profusa
		Olsenella scatoligenes
		Peptostreptococcus stomatis
		Porphyromonas catoniae
		Scardovia inopinata
		Solobacterium moorei
		Streptococcus infantis
		Streptococcus mitis

Supplementary Material 2: Distribution of MetaCyc pathways.

MetaCyc Pathway	Control	Patient
Biosynthesis	190	197
Degradation/Utilization/Assimilation	58	66
Generation of Precursor Metabolites and Energy	42	44
Macromolecule Modification	3	3
Superpathways	8	9

Supplementary Material 3: Distribution of parent pathways.

MetaCyc Pathway	MetaCyc Pathway Class	Control	Patient
Biosynthesis	Amine and Polyamine Biosynthesis	4	5
Biosynthesis	Amino Acid Biosynthesis	37	39
Biosynthesis	Aminoacyl-tRNA Charging	1	1
Biosynthesis	Aromatic Compound Biosynthesis	2	2
Biosynthesis	Carbohydrate Biosynthesis	17	20
Biosynthesis	Cell Structure Biosynthesis	8	9
Biosynthesis	Cofactor, Carrier, and Vitamin Biosynthesis	47	45
Biosynthesis	Fatty Acid and Lipid Biosynthesis	22	23
Biosynthesis	Metabolic Regulator Biosynthesis	1	1
Biosynthesis	Methylerythritol Phosphate Pathways	1	1
Biosynthesis	Nucleoside and Nucleotide Biosynthesis	27	28
Biosynthesis	Other Biosynthesis	2	2
Biosynthesis	Polyprenyl Biosynthesis	2	2
Biosynthesis	Purine Riboucleotide De Novo Biosynthesis	2	2
Biosynthesis	Pyrimidine Ribonucleotide De Novo Biosynthesis	3	3
Biosynthesis	Secondary Metabolite Biosynthesis	7	7
Biosynthesis	Tetrapyrrole Biosynthesis	5	5
Biosynthesis	UDP-N-acetyl-D-glucosamine Biosynthesis	2	2
Degradation/Utilization/Assimilation	Alcohol Degradation	2	3
Degradation/Utilization/Assimilation	Aldehyde Degradation	1	1
Degradation/Utilization/Assimilation	Amine and Polyamine Degradation	3	3

Degradation/Utilization/Assimilation	Amino Acid Degradation	4	5
Degradation/Utilization/Assimilation	C1 Compound Utilization and Assimilation	3	3
Degradation/Utilization/Assimilation	Carbohydrate Degradation	15	18
Degradation/Utilization/Assimilation	Carboxylate Degradation	4	8
Degradation/Utilization/Assimilation	Fatty Acid and Lipid Degradation	1	1
Degradation/Utilization/Assimilation	Hormone Degradation	1	1
Degradation/Utilization/Assimilation	Inorganic Nutrient Metabolism	7	7
Degradation/Utilization/Assimilation	Nucleoside and Nucleotide Degradation	13	13
Degradation/Utilization/Assimilation	Polymeric Compound Degradation	1	1
Degradation/Utilization/Assimilation	Secondary Metabolite Degradation	3	3
Generation of Precursor Metabolites and Energy	Fermentation	17	18
Generation of Precursor Metabolites and Energy	Glycolysis	4	4
Generation of Precursor Metabolites and Energy	Other	1	1
Generation of Precursor Metabolites and Energy	Pentose Phosphate Pathways	3	3
Generation of Precursor Metabolites and Energy	Photosynthesis	3	3
Generation of Precursor Metabolites and Energy	Respiration	2	2
Generation of Precursor Metabolites and Energy	TCA cycle	7	8
Macromolecule Modification	Nucleic Acid Processing	2	2
Macromolecule Modification	Protein Modification	1	1

Supplementary Material 4: **Significant pathway abundance in the species-level (Patient).**

**Significant pathway**	**Average relative abundance patient**
**P125-PWY: superpathway of (R,R)-butanediol biosynthesis**	
g__Streptococcus.s__Streptococcus_gordonii	8.07E-06
g__Streptococcus.s__Streptococcus_sanguinis	2.26E-05
**P185-PWY: formaldehyde assimilation III (dihydroxyacetone cycle)**	
g__Streptococcus.s__Streptococcus_sanguinis	2.36E-05
**RHAMCAT-PWY: L-rhamnose degradation I**	2.33E-05
unclassified	2.33E-05
**PWY-7977: L-methionine biosynthesis IV**	
g__Pseudopropionibacterium.s__Pseudopropionibacterium_propionicum	9.95E-06
g__Lautropia.s__Lautropia_mirabilis	8.17E-06
g__Haemophilus.s__Haemophilus_parainfluenzae	8.06E-06
g__Selenomonas.s__Selenomonas_noxia	5.85E-06
g__Prevotella.s__Prevotella_melaninogenica	4.16E-06
g__Haemophilus.s__Haemophilus_sp_HMSC71H05	4.08E-06
g__Leptotrichia.s__Leptotrichia_wadei	3.63E-06
g__Leptotrichia.s__Leptotrichia_hofstadii	1.99E-06
g__Leptotrichia.s__Leptotrichia_buccalis	1.94E-06
g__Leptotrichia.s__Leptotrichia_sp_oral_taxon_225	1.89E-06
g__Aggregatibacter.s__Aggregatibacter_segnis	1.26E-06
g__Leptotrichia.s__Leptotrichia_sp_oral_taxon_879	1.11E-06
g__Tannerella.s__Tannerella_sp_oral_taxon_HOT_286	1.06E-06
g__Selenomonas.s__Selenomonas_sputigena	8.86E-07
g__Selenomonas.s__Selenomonas_sp_oral_taxon_126	6.95E-07
g__Aggregatibacter.s__Aggregatibacter_sp_oral_taxon_458	6.74E-07
g__Selenomonas.s__Selenomonas_infelix	6.29E-07
g__Prevotella.s__Prevotella_loescheii	4.87E-07
g__Selenomonas.s__Selenomonas_sp_oral_taxon_138	3.88E-07
g__Fusobacterium.s__Fusobacterium_periodonticum	2.73E-07
g__Selenomonas.s__Selenomonas_sp_FOBRC6	1.89E-07

Supplementary Material 5: Significant pathway abundance in the species-level (Control).

Significant pathway	Average relative abundance control
**PWY-6628: superpathway of L-phenylalanine biosynthesis**	
g__Campylobacter.s__Campylobacter_curvus	9.3755E-07
g__Neisseria.s__Neisseria_mucosa	1.27224E-06
g__Selenomonas.s__Selenomonas_sputigena	1.54913E-06
g__Pseudopropionibacterium.s__Pseudopropionibacterium_propionicum	2.7456E-06
g__Selenomonas.s__Selenomonas_artemidis	4.8729E-06
g__Selenomonas.s__Selenomonas_noxia	1.40371E-05
**NAD-BIOSYNTHESIS-II: NAD salvage pathway III**	
NAD-BIOSYNTHESIS-II: NAD salvage pathway III (to nicotinamide riboside)	2.71448E-05
NAD-BIOSYNTHESIS-II: NAD salvage pathway III (to nicotinamide riboside)|unclassified	1.20691E-06
**CITRULBIO-PWY: L-citrulline biosynthesis**	
g__Pseudopropionibacterium.s__Pseudopropionibacterium_propionicum	3.56069E-06
g__Lautropia.s__Lautropia_mirabilis	5.15155E-06
PWY-5100: pyruvate fermentation to acetate and lactate II	
g__Actinomyces.s__Actinomyces_sp_oral_taxon_414	4.45806E-05
g__Fusobacterium.s__Fusobacterium_nucleatum	1.1483E-05
g__Actinomyces.s__Actinomyces_massiliensis	9.78837E-06
g__Streptococcus.s__Streptococcus_gordonii	9.19364E-06
g__Streptococcus.s__Streptococcus_anginosus_group	8.83238E-06
g__Actinomyces.s__Actinomyces_viscosus	6.69078E-06
g__Actinomyces.s__Actinomyces_sp_oral_taxon_897	5.98672E-06
g__Lautropia.s__Lautropia_mirabilis	5.94458E-06
g__Pseudopropionibacterium.s__Pseudopropionibacterium_propionicum	4.02534E-06
g__Streptococcus.s__Streptococcus_oralis	3.91842E-06
g__Actinomyces.s__Actinomyces_johnsonii	3.58296E-06
g__Neisseria.s__Neisseria_macacae	3.48308E-06
g__Actinomyces.s__Actinomyces_naeslundii	2.7594E-06
g__Neisseria.s__Neisseria_flavescens	2.72433E-06
g__Streptococcus.s__Streptococcus_sanguinis	2.29052E-06
g__Streptococcus.s__Streptococcus_cristatus	1.84985E-06
g__Veillonella.s__Veillonella_atypica	1.82114E-06
g__Actinomyces.s__Actinomyces_oris	1.77495E-06
g__Solobacterium.s__Solobacterium_moorei	1.46135E-06

g__Neisseria.s__Neisseria_sicca	1.31422E-06
g__Fusobacterium.s__Fusobacterium_periodonticum	1.30712E-06
g__Veillonella.s__Veillonella_parvula	1.20025E-06
g__Cardiobacterium.s__Cardiobacterium_hominis	1.03712E-06
g__Streptococcus.s__Streptococcus_mitis	8.06433E-07
g__Kingella.s__Kingella_denitrificans	7.4794E-07
g__Neisseria.s__Neisseria_mucosa	6.27943E-07
g__Treponema.s__Treponema_socranskii	6.07718E-07
**PWY-7221: guanosine ribonucleotides de novo biosynthesis**	
g__Actinomyces.s__Actinomyces_sp_oral_taxon_414	7.30496E-05
g__Neisseria.s__Neisseria_flavescens	3.2679E-05
g__Veillonella.s__Veillonella_parvula	3.24552E-05
g__Streptococcus.s__Streptococcus_oralis	2.29819E-05
g__Selenomonas.s__Selenomonas_noxia	1.73789E-05
g__Leptotrichia.s__Leptotrichia_buccalis	1.38209E-05
g__Tannerella.s__Tannerella_sp_oral_taxon_HOT_286	1.3401E-05
g__Gemella.s__Gemella_morbillorum	1.20497E-05
g__Campylobacter.s__Campylobacter_gracilis	1.20457E-05
g__Actinomyces.s__Actinomyces_oris	1.20203E-05
g__Actinomyces.s__Actinomyces_sp_oral_taxon_897	1.19882E-05
g__Streptococcus.s__Streptococcus_anginosus_group	1.17683E-05
g__Aggregatibacter.s__Aggregatibacter_sp_oral_taxon_458	1.16897E-05
g__Selenomonas.s__Selenomonas_artemidis	1.01375E-05
g__Leptotrichia.s__Leptotrichia_wadei	9.00263E-06
g__Neisseria.s__Neisseria_elongata	8.40228E-06
g__Capnocytophaga.s__Capnocytophaga_granulosa	8.21873E-06
g__Fusobacterium.s__Fusobacterium_nucleatum	8.08079E-06
g__Streptococcus.s__Streptococcus_gordonii	7.96563E-06
g__Lautropia.s__Lautropia_mirabilis	7.88291E-06
g__Ottowia.s__Ottowia_sp_oral_taxon_894	7.24237E-06
g__Actinomyces.s__Actinomyces_massiliensis	6.90223E-06
g__Leptotrichia.s__Leptotrichia_sp_oral_taxon_212	6.86686E-06
g__Prevotella.s__Prevotella_melaninogenica	6.77798E-06
g__Porphyromonas.s__Porphyromonas_somerae	6.67363E-06
g__Actinomyces.s__Actinomyces_naeslundii	6.56562E-06
g__Actinomyces.s__Actinomyces_viscosus	6.52623E-06
g__Neisseria.s__Neisseria_mucosa	6.01035E-06
g__Actinomyces.s__Actinomyces_johnsonii	6.00738E-06
g__Aggregatibacter.s__Aggregatibacter_segnis	5.10735E-06
g__Prevotella.s__Prevotella_pallens	4.34417E-06

g__Cardiobacterium.s__Cardiobacterium_valvarum	3.70481E-06
g__Capnocytophaga.s__Capnocytophaga_sp_oral_taxon_332	3.56973E-06
g__Selenomonas.s__Selenomonas_sputigena	3.52604E-06
g__Streptococcus.s__Streptococcus_cristatus	3.49214E-06
g__Capnocytophaga.s__Capnocytophaga_leadbetteri	3.18043E-06
g__Dialister.s__Dialister_invisus	3.09086E-06
g__Haemophilus.s__Haemophilus_parainfluenzae	2.68603E-06
g__Mogibacterium.s__Mogibacterium_diversum	2.67915E-06
g__Veillonella.s__Veillonella_atypica	2.64723E-06
g__Porphyromonas.s__Porphyromonas_sp_oral_taxon_278	2.64449E-06
g__Pseudopropionibacterium.s__Pseudopropionibacterium_propionicum	2.62988E-06
g__Solobacterium.s__Solobacterium_moorei	2.44753E-06
g__Streptococcus.s__Streptococcus_sanguinis	2.41156E-06
g__Eikenella.s__Eikenella_sp_NML130454	2.41019E-06
g__Selenomonas.s__Selenomonas_infelix	2.3968E-06
g__Leptotrichia.s__Leptotrichia_sp_oral_taxon_225	2.37968E-06
g__Prevotella.s__Prevotella_nigrescens	2.30537E-06
g__Selenomonas.s__Selenomonas_sp_F0473	2.29656E-06
g__Neisseria.s__Neisseria_macacae	2.01104E-06
g__Corynebacterium.s__Corynebacterium_matruchotii	1.92814E-06
g__Leptotrichia.s__Leptotrichia_sp_oral_taxon_498	1.89345E-06
g__Cardiobacterium.s__Cardiobacterium_hominis	1.83654E-06
g__Capnocytophaga.s__Capnocytophaga_sputigena	1.59191E-06
g__Streptococcus.s__Streptococcus_mitis	1.56825E-06
g__Campylobacter.s__Campylobacter_showae	1.48031E-06
g__Actinomyces.s__Actinomyces_odontolyticus	1.41912E-06
g__Eikenella.s__Eikenella_corrodens	1.29942E-06
g__Actinomyces.s__Actinomyces_hongkongensis	1.29748E-06
g__Peptostreptococcus.s__Peptostreptococcus_stomatis	1.27028E-06
g__Selenomonas.s__Selenomonas_sp_FOBRC6	1.25194E-06
g__Scardovia.s__Scardovia_inopinata	1.21044E-06
g__Aggregatibacter.s__Aggregatibacter_aphrophilus	1.21043E-06
g__Capnocytophaga.s__Capnocytophaga_ochracea	1.19521E-06
g__Kingella.s__Kingella_denitrificans	1.08356E-06
g__Prevotella.s__Prevotella_oulorum	1.0057E-06
g__Selenomonas.s__Selenomonas_sp_oral_taxon_920	9.45873E-07
g__Campylobacter.s__Campylobacter_curvus	8.81955E-07
g__Capnocytophaga.s__Capnocytophaga_gingivalis	7.90115E-07
g__Neisseria.s__Neisseria_sp_oral_taxon_014	6.96793E-07
g__Selenomonas.s__Selenomonas_sp_oral_taxon_138	6.71305E-07

g__Porphyromonas.s__Porphyromonas_catoniae	6.07353E-07
g__Neisseria.s__Neisseria_sicca	5.83007E-07
g__Olsenella.s__Olsenella_profusa	5.04055E-07
g__Leptotrichia.s__Leptotrichia_sp_oral_taxon_879	4.44253E-07
g__Fusobacterium.s__Fusobacterium_periodonticum	4.30963E-07
g__Rothia.s__Rothia_dentocariosa	3.80698E-07
g__Rothia.s__Rothia_aeria	3.79438E-07
g__Leptotrichia.s__Leptotrichia_sp_oral_taxon_215	3.17243E-07
g__Capnocytophaga.s__Capnocytophaga_sp_oral_taxon_863	2.84675E-07
g__Streptococcus.s__Streptococcus_sp_oral_taxon_056	2.55118E-07
**P41-PWY: pyruvate fermentation to acetate and (S)-lactate I**	
g__Actinomyces.s__Actinomyces_sp_oral_taxon_414	4.45806E-05
g__Fusobacterium.s__Fusobacterium_nucleatum	1.1483E-05
g__Actinomyces.s__Actinomyces_massiliensis	9.78837E-06
g__Streptococcus.s__Streptococcus_gordonii	9.19364E-06
g__Streptococcus.s__Streptococcus_anginosus_group	8.83238E-06
g__Actinomyces.s__Actinomyces_viscosus	6.69078E-06
g__Actinomyces.s__Actinomyces_sp_oral_taxon_897	5.98672E-06
g__Lautropia.s__Lautropia_mirabilis	5.94458E-06
g__Pseudopropionibacterium.s__Pseudopropionibacterium_propionicum	4.02534E-06
g__Streptococcus.s__Streptococcus_oralis	3.91842E-06
g__Actinomyces.s__Actinomyces_johnsonii	3.58296E-06
g__Neisseria.s__Neisseria_macacae	3.48308E-06
g__Actinomyces.s__Actinomyces_naeslundii	2.7594E-06
g__Neisseria.s__Neisseria_flavescens	2.72433E-06
g__Streptococcus.s__Streptococcus_sanguinis	2.29052E-06
g__Streptococcus.s__Streptococcus_cristatus	1.84985E-06
g__Actinomyces.s__Actinomyces_oris	1.77495E-06
g__Solobacterium.s__Solobacterium_moorei	1.46135E-06
g__Neisseria.s__Neisseria_sicca	1.31422E-06
g__Fusobacterium.s__Fusobacterium_periodonticum	1.30712E-06
g__Cardiobacterium.s__Cardiobacterium_hominis	1.03712E-06
g__Streptococcus.s__Streptococcus_mitis	8.06433E-07
g__Kingella.s__Kingella_denitrificans	7.4794E-07
g__Neisseria.s__Neisseria_mucosa	6.27943E-07
g__Treponema.s__Treponema_socranskii	6.07718E-07
